# A Rapid Monitoring and Evaluation Method of Schistosomiasis Based on Spatial Information Technology

**DOI:** 10.3390/ijerph121215025

**Published:** 2015-12-12

**Authors:** Yong Wang, Dafang Zhuang

**Affiliations:** State Key Laboratory of Resources and Environmental Information Systems, Institute of Geographical Sciences and Natural Resources Research, Chinese Academy of Sciences, Beijing 100101, China; zhuangdf@igsnrr.ac.cn

**Keywords:** spatial information technology, remote sensing, geographical information system, schistosomiasis, risk assessment

## Abstract

Thanks to Spatial Information Technologies (SITs) such as Remote Sensing (RS) and Geographical Information System (GIS) that are being quickly developed and updated, SITs are being used more widely in the public health field. The use of SITs to study the characteristics of the temporal and spatial distribution of *Schistosoma japonicum* and to assess the risk of infection provides methods for the control and prevention of schistosomiasis japonica has gradually become a hot topic in the field. The purpose of the present paper was to use RS and GIS technology to develop an efficient method of prediction and assessment of the risk of schistosomiasis japonica. We choose the Yueyang region, close to the east DongTing Lake (Hunan Province, China), as the study area, where a recent serious outbreak of schistosomiasis japonica took place. We monitored and evaluated the transmission risk of schistosomiasis japonica in the region using SITs. Water distribution data were extracted from RS images. The ground temperature, ground humidity and vegetation index were calculated based on RS images. Additionally, the density of oncomelania snails, which are the *Schistosoma japonicum* intermediate host, was calculated on the base of RS data and field measurements. The spatial distribution of oncomelania snails was explored using SITs in order to estimate the area surrounding the residents with transmission risk of schistosomiasis japonica. Our research result demonstrated: (1) the risk factors for the transmission of schistosomiasis japonica were closely related to the living environment of oncomelania snails. Key factors such as water distribution, ground temperature, ground humidity and vegetation index can be quickly obtained and calculated from RS images; (2) using GIS technology and a RS deduction technique along with statistical regression models, the density distribution model of oncomelania snails could be quickly built; (3) using SITs and analysis with overlaying population distribution data, the range of transmission risk of schistosomiasis japonica of the study area can be quickly monitored and evaluated. This method will help support the decision making for the control and prevention of schistosomiasis and form a valuable application using SITs for the schistosomiasis research.

## 1. Introduction

Schistosomiasis is a serious and difficult to eradicate parasitic infection that is widespread at a global level. In 2001, the World Health Organization (WHO) estimated that approximately 200 million people worldwide suffered from schistosomiasis infection and that approximately 600 million people were living in an environment with a risk of infection [1]. Despite all countries actively taking various measures to prevent the spread of infectious diseases and outbreaks, the number of infected individuals or the risk of infection was not significantly reduced [2]. Schistosomiasis is an infectious parasitic disease with local, natural foci of infection. Besides the three general factors affecting infectious diseases (source of infection, route of transmission and susceptible population) which relate to people, schistosomiasis has diverse ways of spreading (e.g*.* more than 40 species of mammals can be infected with *S. japonicum*) [3], which complicate the identification of the source of infection and the control of schistosomiasis. Therefore, the study of a fast and effective method of prevention and control of schistosomiasis is not only a challenge but also essential.

As is known to all, the final hosts of the schistosome are human and animals, and the intermediate hosts are snails. The ecological distribution of schistosome during the growth stage and the geographic distribution of snails appear consistent. Research on the ecological characteristics of snails shows a connection with the epidemiology of *Schistosoma japonicum* [4,5,6]. Snails are distributed predominantly in the grass surrounding lakes, beaches, rivers and irrigation canals. Its distribution is patchy and separate from other areas [7,8]. Because snails are the only intermediate host of *Oncomelania hupensis* and the distribution and survival environment of the snail is the most important factor affecting schistosomiasis, accurate identification of the snail’s breeding ground plays a very important role in recognizing the distribution of schistosomiasis, in preventing and controlling schistosomiasis. Many studies on factors influencing the transmission of schistosomiasis indicate that the distribution of snails is closely related to natural elements such as water distribution, ground temperature (LST), surface humidity (Wetness) and vegetation index (Normalized Difference Vegetation Index, NDVI) [6,9]. Vegetation can keep the soil moist, affect the temperature and shade, and provide abundant food for the snails. Water is necessary for snails’ survival and reproduction. Temperature determines the snails’ rate of growth and development. Suitable temperatures can greatly promote the growth of snails. Humidity also plays a decisive role in the reproduction and distribution of snails. Therefore, the study of the water, vegetation, temperature, humidity and other natural elements is an important aspect of research on the epidemiology of schistosomiasis [9,10,11,12].

SITs are an important technological approach for extracting environmental elements data, such as water, vegetation cover, temperature, humidity [10,13,14]. RS technology has many advantages such as wide coverage, detailed information and repeated observations not affected by the conditions of the geographical environment [11,15]. We can quickly extract information about the ground such as temperature, humidity, vegetation and water based on RS images. While GIS spatial information technology provides tools for the statistical analysis and graphical displaying of spatial variation for the quantitative analysis the factors such as water, vegetation, temperature and humidity [16,17]. GIS is capable of demonstrating not only differences of snails in large (administrative) unit scales but also the distribution and variation of snails on smaller scales (1 km or even hundreds of meters) on an expression grid [18,19], thereby we can use the SITs to study the ecological distribution of snails and assess the risk of schistosomiasis.

Studies on the use of SITs on schistosomiasis have focused primarily on two aspects: (1) from the spatial point of view, the use of RS technology to extract the breeding distribution of snails, accurately calculate the density of snails [8,20,21,22,23], and determine areas of risks of schistosomiasis and (2) through the statistical analysis of spatial correlation combined with GIS technology to estimate the prevalence of schistosomiasis and analyze and forecast the risk areas for schistosomiasis [14,23,24]. Since the advent of SITs, many scholars have studied the ecological distribution of snails and schistosomiasis. As early as 1984, Cross *et al*. used meteorological and RS data (Landsat satellite images) to study and forecast the schistosomiasis endemic areas of The Philippines [25]. In 1999, the British scholar Bavia *et al*. used RS image overlay environmental systems to establish qualitative and quantitative parameters related to spatio-temporal dynamics of schistosomiasis and evaluate the environmental risk factors [26]. Hassan *et al*. used Landsat-TM data and a “Tasseled Cap” method to get geographical characteristic components, such as ground brightness, vegetation and humidity, and, through GIS spatial analysis function, distinguished the incidence of filariasis in 130 villages of the Nile river delta in Egypt; the accuracy of the result was up to 77% [27]. The use of SITs to study schistosomiasis began relatively late in China but has been developing rapidly since then. In 1991, Landsat and NOAA satellite data were used to study the ecological zone division of snails, the infectious sources of schistosomiasis, in the southeast wetlands of China, which provided an important detailed evaluation of the survival environment of schistosomiasis [28]. From then on, RS technologies, GIS spatial analysis function RS data and Bayesian model were combined for forecasting the potential endemic areas of schistosomiasis, space-time distribution, temporal variation and influence [25,29,30,31].

Overall, the study of the control and prevention of schistosomiasis has gradually changed from theoretical research to practical application using SITs. However, some shortcomings still exist: (1) the schistosomiasis forecasting model is still imperfect, and we do not possess fast, dynamic, efficient, orprocess-oriented methods of monitoring and evaluating schistosomiasis over large areas; (2) The combination and use of SITs are neither widespread nor comprehensive. Previous schistosomiasis-related studies only focused on one aspect of the application and could not effectively combine SITs with studies of the monitoring and evaluation of schistosomiasis. In this study, we firstly extract the four geographical characteristic factors—water, vegetation, LST, and ground moisture—and analyze their spatial distribution using SITs, then, explore the ecology of snails and the spatial characteristics of schistosomiasis, which goals are to study a method for rapid monitoring and evaluation of schistosomiasis based on SITs, and evaluate and predict the risk areas of schistosomiasis japonica. This method will greatly shorten the time for identification of potential infectious areas of schistosomiasis, realize the rapid, dynamic, efficient, monitoring and evaluation process of large areas, and provide new ideas and useful methods for the study of ecology and epidemiology of schistosomiasis.

## 2. Materials and Methods

### 2.1. Study Area

**Figure 1:**
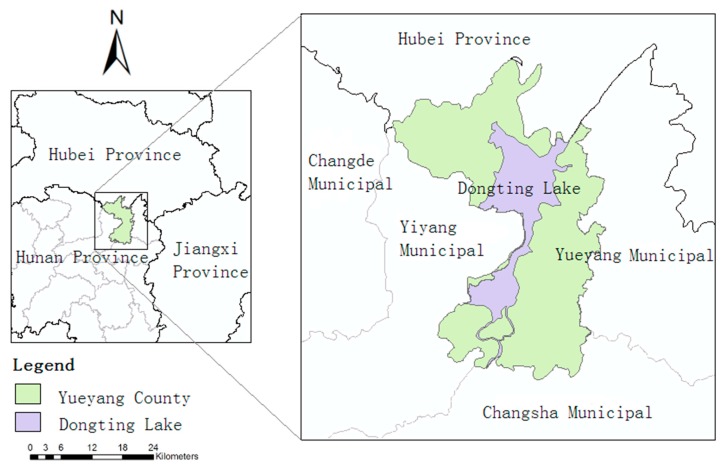
Geographical location of the study area.

In this study, we selected the East Dongting Lake area in Hunan as the study area, as shown in Figure 1. East Dongting Lake, which is the largest lake in the Dongting water system, is located in the Yueyang District, Hunan Province, China. The annual average volume of water is approximately 312.6 billion cubic meters, the water area is 1411 square kilometers, the annual water-storage capacity is 17.8 billion cubic meters, the water depth is 4.22 m, and the maximum water height is 17.76 m. The pH value is 6.8–8.6, suitable for the survival and reproduction of snails. It is a wetland landscape, and the soil is a mixed limnology and river marsh soil. The annual average temperature is 17 °C, the annual average temperature of water is 17.5 °C, and the average annual precipitation is 1200–1330 mm. The east-west length of Dongting Lake is 58 km, and the north-south distance is 76 km. According to the 2012 Hunan Province Statistical Yearbook released by Hunan Statistical Information Network, the total GDP of the Yueyang County is 21 billion RMB, with a population of 72.17 million, of which 28.64 million comprise the urban population and 43.53 million the rural population.

The study area belongs to the east part of the mid subtropical zone and is part of the transition zone, with the winter and summer monsoon winds alternating with each other, due to the combined influence of various factors such as local geomorphic conditions and physical geographic zone. This zone has a sub-tropical monsoon humid climate with heavy continental weather, as well as the characteristics of a mild climate, with four distinctive seasons, rain and heat over the same period, a short cold winter and a long, hot summer. Because of the seasonal water riparian zone, there are many wetland species, especially on the lakeshore grass island, which provides good living conditions for the breeding and reproduction of snails – a typical area for schistosomiasis research.

Dongting Lake is one of the largest schistosomiasis endemic areas in China, with a lake snail breeding area (areas with characteristics suitable for snail breeding) of up to 1721 square kilometers [32]. The snail breeding area of the study area was 978.15 square kilometers, which is 57% of the entire Dongting Lake snail breeding area. Every year in April to October, the Yangtze River is in flood season. The water level of Dongting Lake increases and the surrounding grass land is flooded, in November, after which the water recedes and the beach vegetation is exposed. The schistosome infections of the Dongting Lake usually occur in these bottomlands. People and animals may become infected if they enter the bottomlands and touch the infected water during that time. According to a Chinese schistosomiasis monitoring report, a total of 800,000 patients are infected with schistosomiasis in China: 97.17% of these patients are concentrated in lake regions, and 96.15% of the Chinese snail breeding area is concentrated in lake regions, of which 47.23% are in the Dongting Lake area [12,33].

### 2.2. Data Sources

In this study, we used Landsat TM/ETM image (Resolution 30 m, USGS: Reston, VA, USA) and RapidEye RS image (Resolution 5 m, Kayser-Threde GmbH: Munich, Germany) as the underlying data sources to establish the database of the Schistosomiasis Research Foundation of Yueyang County. Meteorological data came from the website of China meteorological science data sharing service [34]. The Landsat TM/ETM image was used for extracting environment elements and evaluating and deducing risks. The RapidEye RS image was used for sampling the point of observation of the snail density and correcting the density estimation. Two periods of images were used in this research, namely the Landsat TM/ETM RS images of March 2012 and August 2012, with the two RapidEye RS images of the Yueyang district in the same period. The snail data used in this study came from the snail distribution data of East Dongting Lake area and were collected by combining the systematic sampling with the environment random sampling method in March and August, 2012, including the breeding ground position (latitude and longitude), area and density of living snails and their infection rates. The population data used in this study came from the Statistical Yearbook of Yueyang District, Hunan Province.

### 2.3. Overall Process

**Figure 2:**
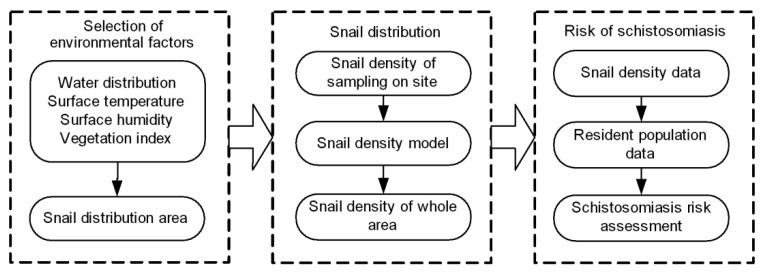
Monitoring and assessment process of schitosomiasis.

The method for the monitoring and evaluation of schistosomiasis based on SITs consists of three main components: selection of environmental factors, snail density analysis and schistosomiasis risk assessment. The overall process of monitoring and evaluation of schistosomiasis is shown in Figure 2.

#### 2.3.1. Extraction of Environmental Factors

The extraction of environmental factors seeks to identify, select and extract the related environmental element data required for the monitoring and evaluation of schistosomiasis, such as extraction of water distribution area, ground humidity, LST, and calculation of NDVI. By extracting the relevant information, and using SITs, we can preliminarily judge the snails’ distribution area.

#### 2.3.2. Estimation of the Density of Oncomelania Snails

Combine the environmental factors data obtaining from RS images during above process with the data of oncomelania snail density sampling on site, the density spatial distribution model of snails could be constructed. Based on the model, we can estimate the snail densities in the whole study area.

#### 2.3.3. Schistosomiasis Risk Assessment

On basis of the distribution and density data of snails in the study area, the regional health risks are evaluated by overlaying the resident population data. Using RS deduction technology and GIS spatial analytical technology, we determine the range of risk of surrounding residents for schistosomiasis.

### 2.4. Selection of Schistosomiasis-Related Environmental Factors

#### 2.4.1. Extracting Water Distribution

Schistosomiasis larvae parasites live in the body of snails. Snails are gastropods belonging to the phylum *Mollusca* whose living environment can be described as “winter on land, summer in water”, meaning that its living environment changes with the season and water temperature [35]. Therefore, extraction water distribution data using RS technology will help us to find the potential breeding ground of snails. This method is mainly based on RS images to recognize and extract information about water. Using RS technology, the methods employed to identify and extract water information usually have included the density slicing method [36], single-band threshold value method [37], multi-band relationship method [38], water index method [39], NDVI method [40], multispectral mixed analysis method [41], RS image classification method [42], and mixed pixel model decomposition method [43]. The density slicing and chromaticity discrimination methods are complex and present a high error rate and low accuracy of water extraction data. The single-band threshold value method is simple and rapid, but its error rate is larger and the accuracy of water identification low. The multi-band relationship method is more accurate than the single-band method, but the band selection and threshold determination process are complex and the implementation poor. The water index method based on multiband spectral information selects several bands closely related to the water characteristics using the mapping relation between the water and the RS spectra, further expanding the resolution of the water and closely related bands to increase the brightness of the water information on the RS image, while the spectrum information of other objects is suppressed to highlight the water. The extraction of water information was achieved using a threshold method. The water index method is widely used because of its high extraction accuracy and strong practicality. The application of the NDVI method, multispectral mixed analysis method, RS image classification method and mixed pixel model decomposition method are complex and require professional knowledge in ecology, RS, and computer science; the method has weak applicability. As a result, we used the water index method to identify and extract water information. NDWI (Normalized Difference Water Index) is the formal name of the water index method [40]. The equation used is:

(1) $$\text{NDWI}=\frac{\text{P}(\text{Green}) -\text{P}(\text{NIR})}{\text{P}(\text{Green}) +\text{P}(\text{NIR})}$$

where P (Green) is the image green band and P (NIR) refers to the near-infrared band.

#### 2.4.2. Ground Temperature Calculation

The most suitable temperature range for both the growth stages of schistosoma and the growth and development of snails was determined. Beyond this range, the growth and development rate of schistosomes and snails are affected. Takahaschi *et al*. [44] found that at a water temperature of 15 degrees C, schistosome larvae exhibit phototropism to any intensity of light, in contrast to temperatures between 15 and 34 degrees C, where the schistosome larvae exhibit light reaction only to certain light intensities. Below 10 degrees C and above 35 degrees C, the schistosome larvae do not exhibit light reaction. The most suitable temperature for the growth of schistosomiasis is 25 degrees C, and the most suitable water pH is 6.6–7.5.

Based on the measured temperature data and RS image data of ground samples, we can quickly calculate the Land Surface Temperature (LST) [45] according to the equation:

(2) $$\text{LST}=\frac{1}{\text{C}}[\text{a}(1-\text{C}-\text{D}) + (\text{b}(1-\text{C}-\text{D}) +\text{C}+ D){\text{T}}_{\text{sensor}}{-\text{DT}}_{\text{a}}]$$

where a = – 67.35535, b = 0.458608, C = ετ, D = (1-τ)[1 + (1-ε) τ], ${\text{T}}_{\text{sensor}}$ is the sensor-measured surface temperature, ${\text{T}}_{\text{a}}\;$ is the average atmospheric temperature, τ is the atmospheric transmittance, and ε is the surface emissivity. Based upon many years of research of the study area’s atmospheric transmittance (τ) and average temperature by Chinese scientists [46,47], we used ε = 0.99, the calculated result is the LST of the water.

#### 2.4.3. Ground Vegetation Index Calculation

Vegetation can not only keep the soil moist, adjust the temperature and shade but can also provide nutrients for snails. The NDVI can reflect adequately the vegetation growth status that describes the ecological environment of the snails and constitute an important indicator to monitor the distribution of snails. NDVI in one of the most widely used vegetation indexes. The equation is [48]:

(3) $$\text{NDVI }=\;\frac{{\text{X}}_{\text{nir}}{-\text{ X}}_{\text{red}}}{{\text{X}}_{\text{nir }}{+\text{ X}}_{\text{red}}}$$

where ${\text{X}}_{\text{nir}}$ is the near-infrared band (0.76–0.90 μm) and ${\text{X}}_{\text{red}}$ is the infrared band (0.63–0.69 μm). The NDVI values range between −1 and +1. A negative value represents no vegetation area such as water, cloud, snow and ice, close to zero represents the soil and rocks, and higher values indicate denser vegetation cover.

#### 2.4.4. Ground Moisture Calculation

A study shows that the snail density in humid areas is much higher than that in dry areas [49]. Therefore, we can calculate the ground moisture of different areas using RS techniques based on RS images. Tasseled cap transformation is a common method for the calculation of ground humidity that uses RS technology [27,50,51]. We obtained the wetness values of snail area from the RS images using the linear tasseled cap method. The equation is [52]:

(4) Wetness = 0.2325CH1 + 0.207CH2 + 0.087CH3 + 0.079CH4 - 0.638CH5 - 0.497CH7

where Wetness is the ground moisture and CH1–CH7 represent the RS images of bands 1–7 of the TM/ETM sensor.

### 2.5. Establishing/Verifying the Snail Density Model

Given that the density and distribution of snails are influenced by natural environmental elements, by extracting the environmental elements associated with the survival of snails-water distribution, surface temperature, ground humidity and ground NDVI, this method can build a regression model in accordance with the snail distribution. The construction of this regression model requires that the dependent variables are independent and fit the normal distribution and that the independent variables are not random variables. The model is usually a simple linear correlation [52]. Because the complex distribution data of snails cannot be fit with a simple linear regression model, in this study, we built a generalized linear mixed model for exponential variables by removing the assumption limit on the dependent variable normality of the classical linear regression model to construct a snail density estimation model [53]. The equation is:

(5) $${\text{D}=\text{g}}^{-1\;}{(\eta ) =\text{g}}^{-\;1}{(\text{NDVI}\times \beta }_{1}{+\text{ LST}\times \beta }_{2}{+\text{ Wetness}\times \beta }_{3}{+\text{ Distancex}\beta }_{4}+\text{Z}\gamma ) +\varepsilon$$

where D represents the density of snails, ${\text{g}}^{-1\;}()$ is the inverse function of the monotonic differentiable continuous function g(), β is the estimated parameters of the corresponding observed variables, NDVI represents the NDVI, LST represents the ground temperature, Wetness represents the ground humidity, Distance represents the water distance, Z represents a random variable, γ represents the estimated parameters corresponding to the random variable, and ε is the random error.

According to the characteristics of the sample data in this study, we use cross validation method to verify the results of the model. That is dividing the sample data into training set and validation set for constructing the snails’ density model and checking the model validation respectively. Firstly, the training set is used for training the model and modeling. Then we use the validation set to verify the accuracy of the model, and use coefficient of determination (*R*^2^) and Root Mean Square Error (RMSE) to evaluate the accuracy of the model. 

Coefficient of determination:

(6) $${\text{R}}^{2}\text{ = }\frac{{{(\text{Y}}_{\text{p}}-\bar{{\text{Y}}_{\text{p}}})}^{2}}{{\left(\text{Y}-\bar{\text{Y}}\right)}^{2}}$$

where Y and $\bar{\text{Y}}$ are the original value and its average value of the training set, respectively. Y_p_ and $\bar{{\text{Y}}_{\text{p}}}$ are the original value and its average value of the validation set, respectively.

Root Mean Square Error:

(7) $$\text{RMSE = }\sqrt{\frac{\sum {\left(\text{Y}-{\text{Y}}_{\text{p}}\right)}^{2}}{\text{n}}}$$

where Y is the original value of the training set, $\bar{{\text{Y}}_{\text{p}}}$ is the original value of the validation set, *n* is the sample size. Much bigger *R*^2^ and/or much smaller RMSE show that the model has more stability and/or more accuracy.

### 2.6. Evaluation of Schistosomiasis Risk Area

**Figure 3:**
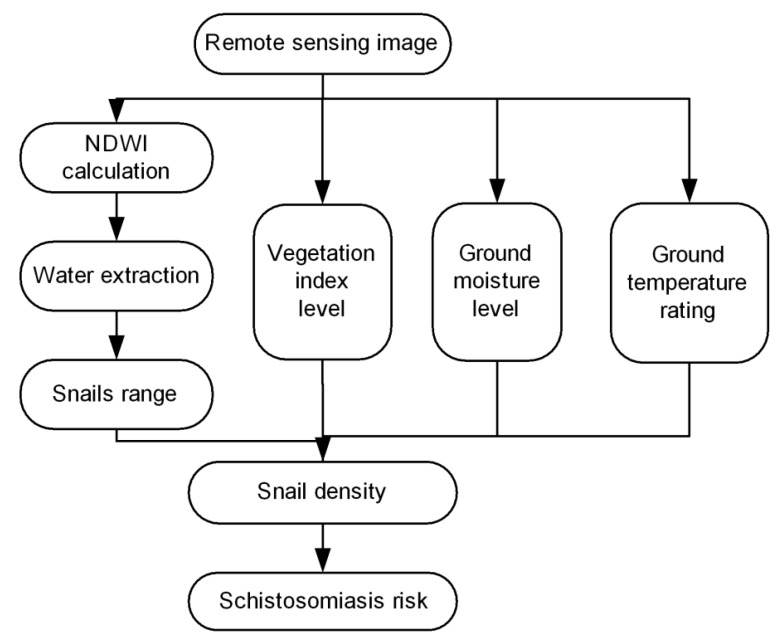
Schistosomiasis risk area assessment process.

Schistosomiasis risk assessment is to use GIS spatial analysis method and remote sensing deduction method to determine the risk area of schistosomiasis by: (1) extracting or computing elements information related to snail breeding environment from RS data; (2) calculating the density of oncomelania and (3) overlaying ground temperature risk area, ground humidity risk area and population density data. The process was performed as follows (Figure 3):

Step 1: Extracting water area. Using the band algorithm Equation 1 to calculate autumn-winter and summer NDWI using corresponding RS images and extract water area, where NDWI is greater than 0.05.

Step 2: Ascertaining the potential snail breeding area. Using band subtraction algorithm to extract the difference between two water images, that is “Water difference area” and the “winter on land, summer in water” areas. Then, ascertain the potential snail breeding area in the light of the corresponding characteristics.

Step 3: Ascertaining each level risk areas of snail breeding area. Because the snail breeding area was flooded in the wet season and exposed only in the dry season, we use Equation 3 to calculate the NDVI value of the dry season image. We then extracted the areas where NDVI values are greater than 0.2 and ascertain each level risk of snail breeding area.

Step 4: Confirming the accurate snail breeding area. We use the image overlay algorithm to obtain the intersection of the areas of two images where both met Steps 2 and 3. The result is the more accurate snail breeding ground.

Step 5: Confirming the LST risk area. We use Equation 2 to calculate the LST. For 20 to 25 degrees C, the breeding snail optimum temperature, the areas that have temperatures closer to 20 to 25 degrees C are set as the greater risk areas.

Step 6: Confirming the ground humidity risk area. The risk areas are divided into different levels according to the ground humidity Equation 4.

Step 7: Confirming the schistosomiasis risk area. We use Equation (5) to calculate the snail density. Further on, we use GIS spatial analysis method and remote sensing deduction method to overlay the LST risk area, the ground humidity risk area and the population distribution data to ultimately determine the risk of schistosomiasis.

## 3. Results and Discussion

### 3.1. Result of Extraction of Environmental Element

**Figure 4:**
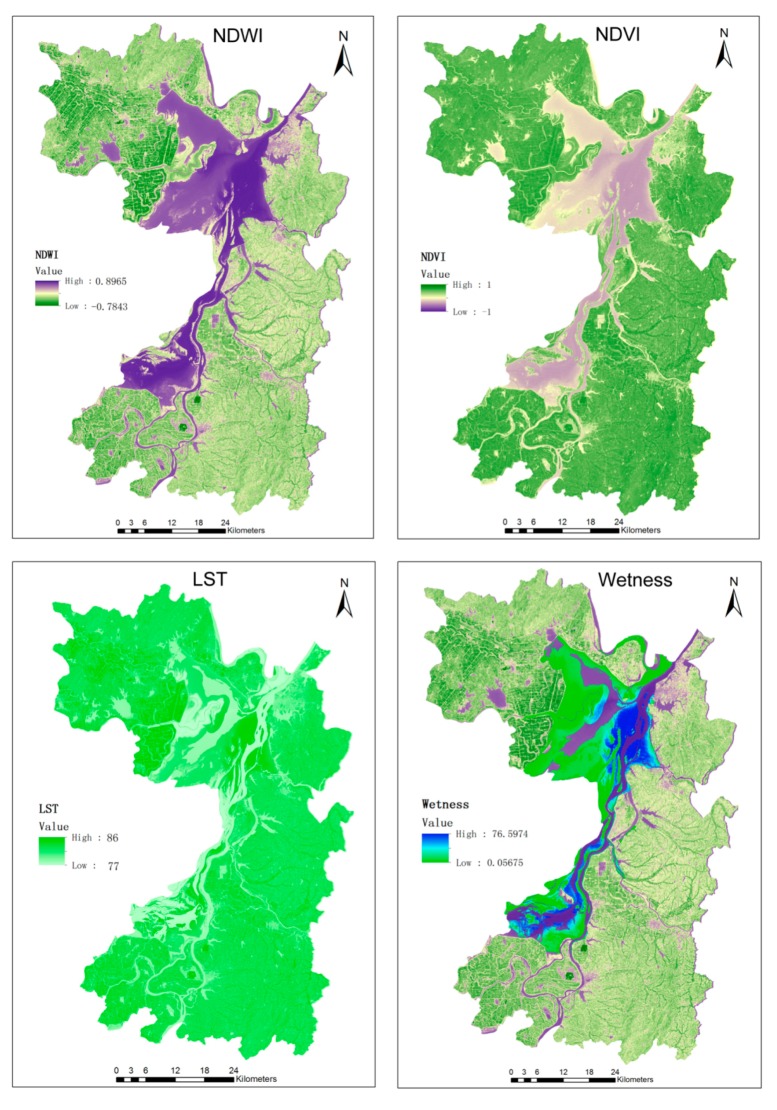
Extraction of environmental element data from remote sensing images.

Using RS technology, NDWI, NDVI, LST and Wetness were calculated according to Equations (1)–(4) as shown in Figure 4. According to the images, the calculated average NDVI of the Dongting Lake area in Yueyang County, Hunan Province, in 2012 was 0.453, the LST was 17.5, and the Wetness was 31.3. The average distance between the study point and the Dongting Lake water d was 115 m.

**Figure 5:**
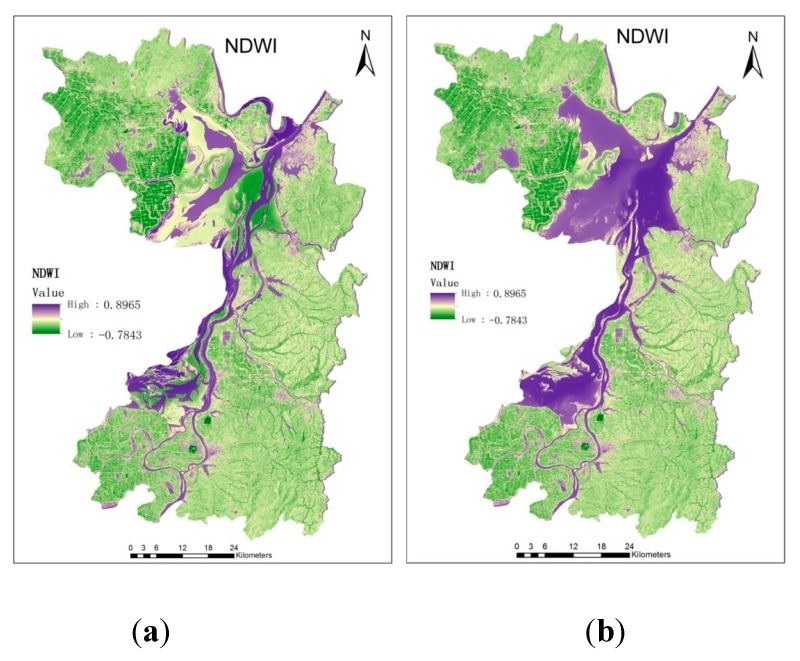
NDWI variation map during the dry season in March (**a**) and the raining season in August (**b**).

By extracting the water distribution of Dongting Lake, we found that during the dry season from November to March, the northern part of the Dongting Lake turned to a narrow waterway. April to October comprised the wet season, during which the northern part of Dongting Lake became wider. The water distribution map during the March and August dry season, which was extracted using RS images, is shown in Figure 5.

As shown in Figure 5, the water level dropped in the dry season, and large areas of bottomland and grassland were exposed. We can speculate that these areas are the living areas of snails, the schistosome host. Using the GIS space statistical tools, we calculated that during the wet season (August), the East Dongting Lake water area was 1,411 square kilometers. During the dry season (March), the water area was only 433 square kilometers, a decline of 69%, whichwas composed of 978 square kilometers of bottomland.

### 3.2. Results of Snail Density Calculation and Relative Risk Evaluation of Schitosomiasis

**Figure 6:**
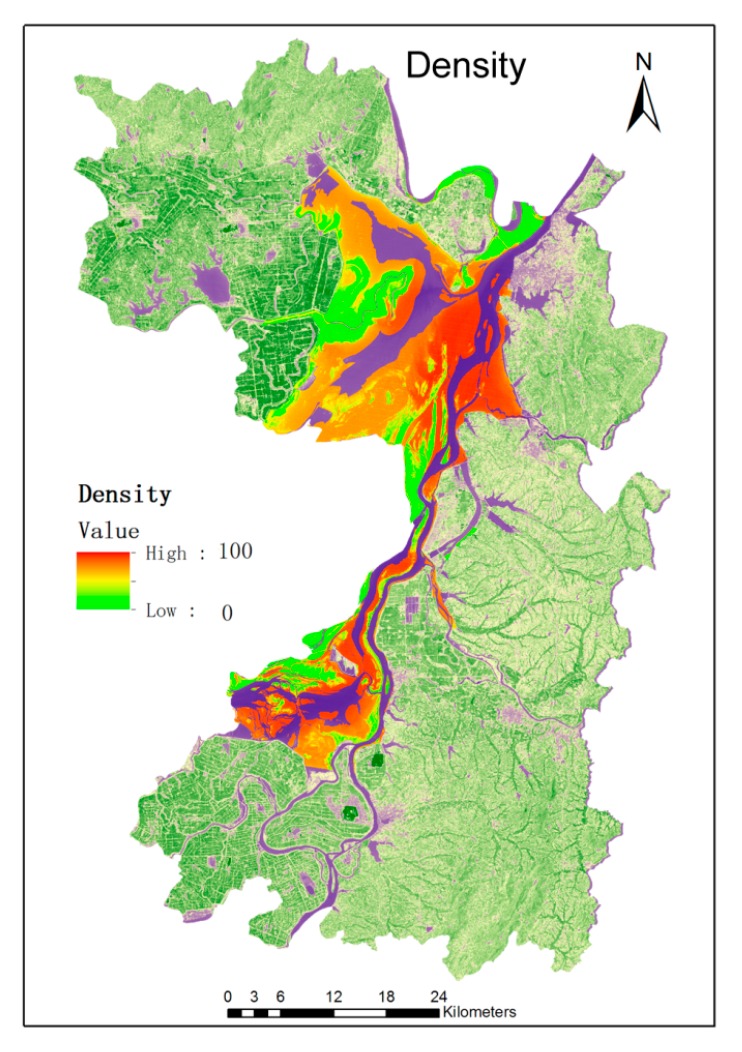
The predicted snail distribution.

Combined with the ground humidity, LST and NDVI in the study area, the spatial distribution of the predicted density of snails is shown in Figure 6. As shown in Figure 6, the areas with the highest risk of snail density distribution are the eastern and southern sections of East Dongting Lake. Areas surrounding the northeastern part of Dongting Lake, such as Matang Town, Antlers Town, and Qu Yuan Farm have the highest density of snails. Westward along the Dongting Lake, the snail density decreased gradually.

Using the generalized linear mixed model Equation (5), we can construct the snail density model based on the data of environmental elements and the data of *Oncomelania hupensis* sampling. After verifying by using the validation Equations (6) and (7), the final models of the snails in the Dongting Lake and the environmental elements are:

(8) D1 = -0.005784d - 0.2256t + 1.954m - 0.874v + 16.753

(9) D2 = 0.0774d + 0.1869t + 0.0857m - 0.5473v - 4.832

where D1 and D2 represent the density of snails in the dry season (March) and wet season (August) in the study area, respectively. d represents the distance between the observation point and water, t represents the surface temperature (LST), m represents the ground humidity (Wetness), and v represents the NDVI (NDVI).

Using GIS and RS technology, combined with the sampling data, we calculated each risk level of the breeding area and the density of snails, as shown in Table 1.

**Table 1:** Risk level of the study area and density of breeding snails.

Level	Area	Density of Breeding Snails (One/0.11 m^2^)
1	2.93 km^2^	>27
2	3.12 km^2^	18~27
3	1.06 km^2^	9~18
4	0.94 km^2^	1~9
5	1.81 km^2^	<1

**Figure 7:**
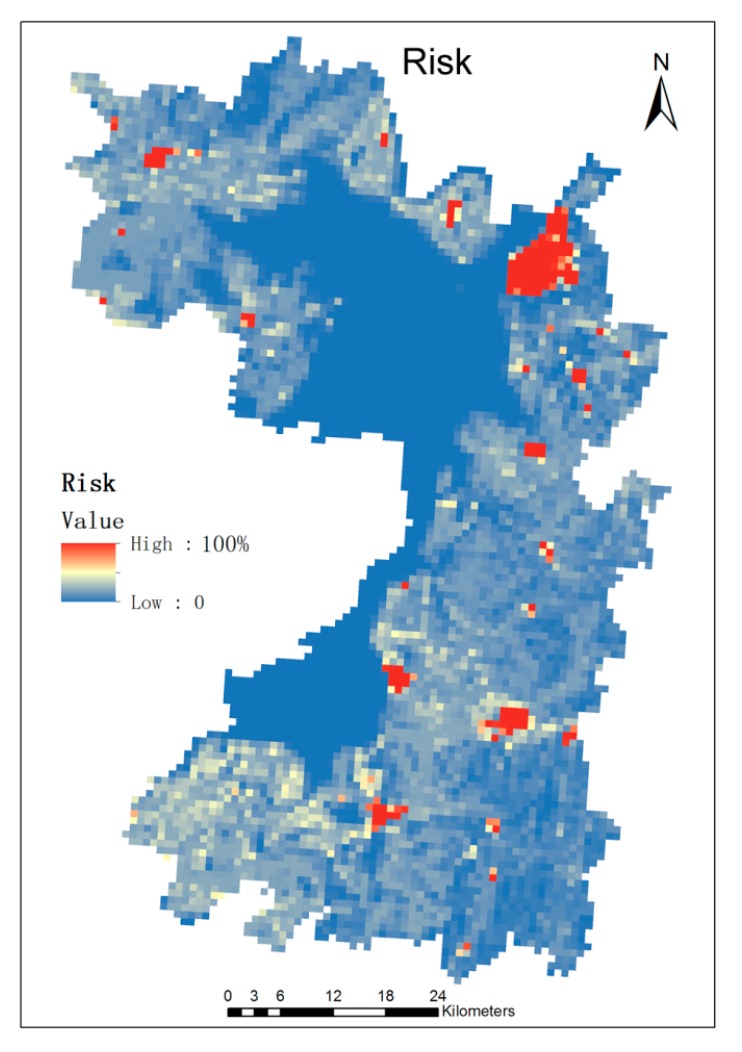
Map of the relative risk of schistosomiasis.

Using the snail density data, combined with the population data of the study area and RS deduction and GIS spatial analysis technology, we calculated the risk of schistosomiasis in the study area, as shown in Figure 7.

The schistosomiasis infection rate of each town in the study area was divided into four grades: 0% to 10%, 10% to 25%, 25% to 50% and 50% to 75%, of which the highest risk of schistosomiasis was in Matang town, Liu Linzhou town, Antlers town, Meixi town and Quyuan farm. The risk of schistosomiasis infection in these towns was up to 50%–75%, highlighting the need to focus on the prevention and control of the risk of schistosomiasis infection. Linzhai town, Gutang town, Xianglin town and Santang town followed with a risk of schistosomiasis infection that reached approximately 25% to 50%.

Generally speaking, the larger the density of snails there is, the higher the risk of infection of the surrounding residents will have. The longer the distance away from Dongting Lake is, the lower the risk of infection of schistosomiasis they will have. However, in some towns such as Leiluo town and Dongtang town, which are located far from Dongting Lake, the risk of schistosomiasis infection was also very high, probably because of their ditch density and rich landscape heterogeneity. The surrounding residents live close to the ditch pond, leading to the increased risk of schistosomiasis infection.

### 3.3. Discussion

#### 3.3.1. Extraction of Environmental Elements

In this paper, by using the field data combined with RS technology to extract the environmental elements such as the water distribution, NDVI, surface temperature and ground humidity, we aimed to predict the density of oncomelania snails, and then combine population distribution data to predict the transimission risks of the disease.

Our study showed that for a small range area of water, the density of oncomelania snails was positively correlated with surface moisture. In a wider surface area of water, the oncomelania snails density was positively correlated with NDVI. In small range areas of water, low NDVI and high humidity showed that these areas have rich water content and may be more suitable for the survival of snails. In wider range areas of water, the increase in NDVI showed rich vegetation cover, providing more potential breeding environment for snails. Therefore, using RS images can quickly identify the living areas of snails. Using RS images as data sources and monitoring the snail distribution at different water levels, we found that a stable water level will cause the oncomelania snails density to be at a relatively low average and variation level. In contrast, rapid changes in water level will cause the snail density to reach a higher variation level. Similarly, changes in temperature and humidity will affect the survival condition of snails. There are different degrees of correlation between temperature, humidity, water changes and NDVI that have strong correlations with the density of oncomelania snails and show obvious lag effects.

Due to the interactions between the survival environmental elements of snails, effectively calculating the density of oncomelania snails by any snail density calculation model based on a single environmental factor is very difficult, but spatial information technology such as RS and geographic information system can easily reveal the space-time relation between those environment elements [54,55,56]. Using spatial information technology to extract the environmental elements related with the breeding environment of snails is an effective method.

#### 3.3.2. Comprehensive Assessment

The present study combines the temperature, humidity, water change and NDVI into a common environmental suitability index. Representing the environmental balance status, which is determined using the water, temperature, humidity and NDVI, hints as to whether the environment is suitable for the breeding of snails. This index has obvious seasonal trends: it is high in the summer because of the high water level, high temperature and high NDVI and low in the winter because of the low water level, low temperature and low NDVI. In the spring and autumn, the water level, temperature and vegetation will be in balance, and thus, the index will reach a suitable value.

The relationship between a suitable snail breeding environment index and the density of snails shows that in the same month, there is a positive linear relationship between the snail breeding environmental suitability index and snail density. A positive linear relationship and cubic curve non-linear relationship exist between the environmental suitability index of one month and the density of snails the following month. Primarily, the influence of the environmental suitability index on the snail density displays a hysteresis effect; index values affect the density of snails, with obvious seasonal trends. In the summer and winter, with the loss of balance between temperature and humidity, when the absolute value of the index is higher (>1) or lower (near 0), the environment will limit the breeding and reproduction of snails, causing the density of snails to decrease. In the spring and early autumn, the absolute value of the temperature humidity index is at a moderate level (0~1); the temperature and humidity are in balance, the environment is suitable for the breeding of snails, and the variation of snail density shows a crest, consistent with the actual changes in snails. Using actual statistical sampling, we found that there was no obvious correlation between the density of snails and NDVI or humidity, and we showed that the impact of the snail distribution is obviously different. We speculate that the degree of influence of social and economic factors (e.g. household income), population, number of livestock, livestock rearing methods and sanitary condition are stronger than that of natural factors.

#### 3.3.3. Key Factors

Natural environmental elements are important factors affecting the distribution of snails. The correct analysis of the spatial characteristics of the distribution of snails and the study of the relationship between natural environmental elements and snail distribution play a very important role in the control of snails and the prevention of schistosomiasis. Meanwhile, human and social factors are also important factors affecting the risk of schistosomiasis. Many aspects of social factors are reflected in human effects on environment and land use information, which are closely related to human activities, such as settlement distribution, canal distribution, and types of crops and livestock distribution. Therefore, in addition to parameters such as water distribution, NDVI, temperature, and humidity, the method of landscape pattern analysis based on information on land use and land cover types is also an important technical approach for monitoring the distribution of snails. Many factors affect the snails, and there is a certain correlation between these factors. The degree of influence of different factors on the snail distribution is also different and depends on the spatial scale. On a large scale, the main factors affecting the snail distribution include LST and rainfall, among others. However, on a small scale, the influential factors are the water distribution, NDVI, residential distribution, and landscape pattern, among others. Bayesian space-time dynamic monitoring of the snail distribution shows that as time changes, the density of oncomelania snails also changes according to time and space.

Some aspects of human factors such as custom, habits, and cultural background may affect the intensity of the spread of schistosomiasis. Epidemics and spread of *Schistosomiasis japonica* are affected not only by the susceptibility level of the schistosomiasis infection risk area but also by the risk of schistosomiasis infection caused by the combination of the strength of contact between human and infected water and the susceptibility level. Current research [57] shows that work contact of adult males with infected water is the main source of contact, as is the labor contact and life contact of adult females, as well as the playful contact of children and teenagers. Our research predicts the risk areas and divides them into different levels. To reduce the risk of schistosomiasis infection risk during the working period, it would be important to prohibit workers from working in high-risk areas and to prohibit teenagers from playing in the affected areas. Economic factors are also key risk factors for the transmission of schistosomiasis: improving the economic level such as a wide spread of water facilities and using machine production instead of cattle production can greatly reduce the labor contact with the affected water and reduce the risk level.

## 4. Conclusions

The purpose of our research on the spread of schistosomiasis and the RS monitoring of the schistosomiasis epidemic environmental elements was to monitor the risk of spread of schistosomiasis in the epidemic areas and provide decision support for the establishment of related policies. Using SITs to monitor and evaluate schistosomiasis has incomparable advantages over simple statistical investigations. We analyzed the extraction of environmental elements related to schistosomiasis from RS images, the constructing method of snail density prediction model, and the process evaluation of schistosomiasis in the manuscript. Our study showed that extraction and calculation of environmental elements, such as water, temperature, humidity, NDVI, in Dongting Lake, Yueyang County, Hunan Province is an important and necessary prerequisite for calculating the density of snails. By monitoring and evaluating models, we can quickly and efficiently calculate the range and density of the snail breeding ground and evaluate the risk of schistosomiasis. Providing the technical support for the monitoring and evaluation of schistosomiasis is a feasible technical method.

By using RS images to extract data on water, humidity, LST, and calculate NDVI, to establish the relationship between the natural environmental elements and snaildensity using GIS, through the finite snail density sampling data, the inverse snail density model for a large area combined with the RS data, finally completed the risk assessment for schistosomiasis, SITs play a key role. We presented a schistosomiasis surveillance assessment method based on SITs in the paper, which will provide important support for monitoring and control of schistosomiasis, reducing the potential for infection area of schistosomiasis identification time, realizing dynamic, efficient, fast and large area monitoring and evaluation of schistosomiasis, what is a positive and useful exploration application using SITS in the study of schistosomiasis epidemiology.

Of course, we recognize that our rapid method has possible limitations. For example, we focused on the nature influencing factors whereas we ignored some influence of social and economic factors (e.g. household income), sanitary conditions, *etc*. In the future, we must improve our methodology to consider most of the influencing factors on schistosomiasis.

## Supplementary Files

Supplementary File 1
